# A CNN Sound Classification Mechanism Using Data Augmentation

**DOI:** 10.3390/s23156972

**Published:** 2023-08-05

**Authors:** Hung-Chi Chu, Young-Lin Zhang, Hao-Chu Chiang

**Affiliations:** Department of Information and Communication Engineering, Chaoyang University of Technology, Taichung 41349, Taiwan

**Keywords:** sound classification, signal processing, CNN

## Abstract

Sound classification has been widely used in many fields. Unlike traditional signal-processing methods, using deep learning technology for sound classification is one of the most feasible and effective methods. However, limited by the quality of the training dataset, such as cost and resource constraints, data imbalance, and data annotation issues, the classification performance is affected. Therefore, we propose a sound classification mechanism based on convolutional neural networks and use the sound feature extraction method of Mel-Frequency Cepstral Coefficients (MFCCs) to convert sound signals into spectrograms. Spectrograms are suitable as input for CNN models. To provide the function of data augmentation, we can increase the number of spectrograms by setting the number of triangular bandpass filters. The experimental results show that there are 50 semantic categories in the ESC-50 dataset, the types are complex, and the amount of data is insufficient, resulting in a classification accuracy of only 63%. When using the proposed data augmentation method (*K* = 5), the accuracy is effectively increased to 97%. Furthermore, in the UrbanSound8K dataset, the amount of data is sufficient, so the classification accuracy can reach 90%, and the classification accuracy can be slightly increased to 92% via data augmentation. However, when only 50% of the training dataset is used, along with data augmentation, the establishment of the training model can be accelerated, and the classification accuracy can reach 91%.

## 1. Introduction

Sound refers to the physical vibration or disturbance that travels through a medium, such as air or water, and can be perceived by the human ear or other auditory systems. It is a broader concept that encompasses all audible vibrations, including those occurring in nature, produced by musical instruments, or generated by man-made sources. Sound is a natural phenomenon that exists regardless of whether it is captured or recorded. On the other hand, audio is the electrical or digital representation of sound that has been captured or recorded. It specifically refers to the electronic representation or signal that represents sound waves and can be stored, transmitted, or reproduced. Audio can be analog or digital, and can be stored in various formats, such as WAV, MP3, or FLAC. It is typically used to refer to the processed or recorded sound that can be played back or manipulated using audio equipment or software.

Sound is a complex, feature-rich signal, and sound classification is receiving strong interest in a growing number of application areas, from speech recognition [1,2], music analysis and recommendation [3,4], environmental sound monitoring [5,6], and anomaly detection and security [7,8].

- Speech recognition [1,2]: Sound classification plays a crucial role in speech recognition systems. By accurately classifying and identifying speech sounds, these systems can convert spoken words or phrases into written text. This technology is utilized in voice assistants, transcription services, call center automation, and language learning applications. Accurate sound classification enables more precise and efficient speech recognition, leading to better user experiences and increased productivity.
- Music analysis and recommendation [3,4]: Sound classification allows for the analysis and categorization of music based on various features, such as genre, tempo, mood, and instrumentation. This classification enables personalized music recommendations, playlist generation, music organization, and automatic tagging. Music-streaming platforms and digital music libraries rely on accurate sound classification to provide personalized recommendations to users, enhancing their music listening experiences.
- Environmental sound monitoring [5,6]: Sound classification can be used to monitor and classify environmental sounds. This is useful in applications such as wildlife monitoring, noise pollution assessment, acoustic event detection, and surveillance systems. By automatically classifying sounds such as animal calls, vehicle sounds, alarms, or gunshots, sound classification aids in detecting anomalies, identifying specific events, and alerting authorities or users to potential threats or disturbances.
- Anomaly detection and security [7,8]: Sound classification can be used to identify abnormal or anomalous sounds in various contexts, including industrial settings, security systems, and healthcare environments. By training models to recognize normal sound patterns, deviations or unexpected sounds can be classified as anomalies. This technology helps to detect equipment failures, security breaches, and medical emergencies, allowing for timely interventions and preventive measures.

However, sound classification methods relying on signal-processing techniques involve various steps to extract relevant features and classify sound signals. Initially, the raw, unprocessed sound signal, which is electronically captured and recorded and stored in a digital format, is called an audio signal. Then, audio signals are preprocessed by eliminating noise, normalizing amplitudes, and segmenting into smaller frames. Feature extraction is performed by analyzing the frequency content of these frames using techniques such as the Fourier transform or the short-time Fourier transform (STFT). The resulting frequency domain representations can be further processed to obtain features such as spectral energy, spectral centroid, or spectral roll-off. Additional temporal features such as the zero-crossing rate and time-domain statistical measures (e.g., mean, variance) can also be computed. Finally, these extracted features are used to train a classifier, often based on statistical models such as hidden Markov models (HMMs) or Gaussian mixture models (GMMs). Signal-processing-based methods rely on careful feature engineering and domain expertise but may face limitations in handling complex patterns or generalizing to unseen data, compared to more advanced techniques such as deep learning.

The existing sound classification methods face several challenges and limitations, including:

- Cost and resource constraints: Collecting and labeling sound data can be a time-consuming and resource-intensive process, which requires expertise, equipment, and human effort to capture, process, and accurately annotate sound signals. The cost and logistics associated with data collection and annotation can be significant, especially when aiming for large-scale and diverse datasets. Limited financial resources and access to specialized equipment or personnel can pose challenges in acquiring an adequate amount of labeled sound data.
- Data imbalance: Imbalanced class distributions within sound datasets can represent another obstacle. Certain sound classes may have an abundance of available data, while others are underrepresented. This data imbalance can negatively impact the model’s performance, as it may struggle to generalize well for minority classes with limited examples. Acquiring a balanced dataset with sufficient instances for each class becomes a challenge, leading to potential biases and a reduced classification accuracy for certain categories.
- Data annotation challenges: Accurate labeling of sound data is a complex task that often requires human expertise and domain knowledge. Annotating sound signals with the correct class labels or semantic information can be subjective and prone to errors. The process may involve multiple annotators, leading to variations in the annotations and potential inconsistencies. The scarcity of well-annotated sound data further hinders the acquisition of enough labeled samples for classification.

To address these challenges, researchers often combine signal-processing techniques with machine learning approaches, such as using deep learning models, to automatically learn relevant features from sound data. This combination of methods aims to overcome the limitations of traditional signal processing and improve the accuracy and adaptability of sound classification systems. This is due to the ability of deep learning models to learn complex patterns and extract discriminative features from sound data, resulting in an improved accuracy and the ability to distinguish similar sound classes. They exhibit robustness to variability by effectively handling diverse acoustic environments and speaker and instrument variations, and they can generalize well to unseen data. Furthermore, deep learning models can efficiently scale to process large-scale sound datasets in real time or near real time, enabling rapid analysis. They can leverage unlabeled data through unsupervised or semi-supervised learning techniques, effectively capturing valuable information and improving the classification performance, even in scenarios with limited labeled data availability. Advancements in interpretability techniques also make it possible to gain insights into the decision-making process of deep learning models, addressing concerns related to the transparency and explainability. Overall, deep learning methods hold great promise in overcoming the limitations of existing sound classification approaches, paving the way for more accurate and robust sound analysis.

Therefore, this paper proposes the use of a CNN model [9] for sound classification. The sound signals in the dataset are preprocessed using the Mel-Frequency Cepstral Coefficients (MFCCs) method, and appropriate parameter adjustments are made for data augmentation. Data preprocessing is utilized to develop a CNN classification model, addressing issues such as cost and resource constraints, data imbalance, and data annotation problems. The rest of this paper is organized as follows: In Section 2, we analyze related work proposed by other authors in detail, sort out the advantages and disadvantages of their proposed methods, and further clarify our research goals. In Section 3, we clearly explain our proposed sound classification model and data augmentation method using MFCCs. In Section 4, we present experimental results simulated under different sound datasets with high classification accuracy and data augmentation practicability, and in the last section, we conclude the paper based on our proposed methods and summarize their performance breakthroughs, while also presenting a brief overview of our future research.

## 2. Related Works

Sound represents the vibration density of an object in the air that changes over time. If this signal is to be stored and analyzed, it must first be digitized [10]. Sound includes three elements: volume, pitch, and timbre. Volume refers to the loudness of the sound, which is affected by the amplitude of the sound wave. The higher the amplitude, the higher the volume of the sound waveform, measured in decibels (dB). Pitch refers to the highness or lowness of a sound and is influenced by the frequency of the vibration of the sound wave. The higher the fundamental frequency of the sound, the higher the pitch, measured in hertz (Hz). Timbre refers to the quality or content of a sound and is represented by the variation of each waveform within a fundamental period. Different timbres represent different sound contents, such as different letters having different pronunciations, all due to variations in timbre.

Traditional sound classification methods that rely on signal-processing techniques involve various steps to extract relevant features and classify sound signals. Initially, sound signals are preprocessed by eliminating noise, normalizing amplitudes, and segmenting into smaller frames. In the process of feature extraction, the frames of audio signals are analyzed to examine their frequency content using techniques like the Fourier transform [11] or the short-time Fourier transform (STFT) [12]. These analyses produce frequency domain representations that can be further processed to obtain features, including spectral energy, spectral centroid, and spectral roll-off. Additional temporal features like the zero-crossing rate and time-domain statistical measures can also be computed. Subsequently, the extracted features are utilized to train a classifier, typically employing statistical models such as hidden Markov models (HMMs) [13] or Gaussian mixture models (GMMs) [14]. Traditional signal-processing-based methods rely on careful feature engineering and domain expertise but may face limitations in handling complex patterns or generalizing to unseen data, compared to more advanced techniques such as deep learning.

Therefore, in the volume application of traditional sound classification, the sound signal can be evaluated according to the location of the source of the sound and the amplitude [15]. However, since the transmission of the volume will decrease with the increase in the transmission distance, the distance between the sound-emitting point and the sound-collecting point may result in different volumes of signals of the same sound type. In the pitch application of traditional sound classification [16], factors such as pitch, long-term average spectrum, formant, and noise components are considered to develop the classification of singing voices to distinguish soprano, alto, tenor, and bass. In [17], a feature extraction method was proposed for the classification of environmental sound events based on time–frequency representation.

In the field of sound processing, Mel-Frequency Cepstrum (MFC) [18] is a linear transformation of the logarithmic energy spectrum based on the nonlinear Mel scale of sound frequency. Mel-Frequency Cepstral Coefficients (MFCCs) are the coefficients that make up the MFC. They are derived from the cepstrum of an audio clip. The difference between the cepstrum and the MFC is that the frequency band division of the MFC is equally spaced on the Mel scale, which is more approximate to humans than the linearly spaced frequency bands used in the normal log cepstrum auditory system. The processing of MFCCs includes frame blocking, pre-emphasis, a Hamming window, signal transformation, a Mel filter, and discrete cosine transform (DCT). These steps will be described in detail in Section 3.1.

With the rapid development of artificial intelligence technology, deep learning has become one of the important technologies to solve problems in various fields. Deep learning [19] has emerged as a powerful technique for sound classification, demonstrating remarkable performance in various applications. Deep learning models, such as convolutional neural networks (CNNs) [20] and recurrent neural networks (RNNs) [21], can automatically extract relevant features directly from raw sound waveforms, enabling more effective representation learning. These models can capture low-level acoustic features, such as spectral patterns and temporal dynamics, as well as higher-level semantic information, allowing them to discern intricate sound characteristics and classify sound signals with high accuracy. By training deep learning models on large-scale annotated sound datasets, they can generalize well to unseen sound samples and exhibit robust performance across various sound classification tasks, including speech recognition, music analysis, environmental sound monitoring, and more. The flexibility and adaptability of deep learning make it a promising approach toward advancing sound classification capabilities and unlocking new possibilities in sound-related applications.

All kinds of deep learning have their own characteristics and suitable application fields. Among them, CNN has a good effect on image classification, and its model includes a convolutional layer, a pooling layer, and a flat layer. The convolutional layer is used to preserve the spatial structure in the picture and extract features from such a structure, the pooling layer is used to reduce the parameters of the neural network and reduce the computational cost while maintaining the feature invariance, and the fully connected layer is the output layer, using the SoftMax function to output classification results. In [22], a new deep convolutional neural network is proposed for sound classification, which uses a concatenated spectrogram as input features to increase the richness of features. It is generated by concatenating the Log-Mel and the Log-Gammatone spectrograms. The proposed method was tested in the datasets ESC-50 [23] and UrbanSound8K [24], and the classification accuracies were 83.8% and 80.3%, respectively. In [25], an ACDNet based on adaptive combined dilated convolutions is used, and a general pipeline is proposed that can automatically convert large, deep convolutional networks through compression and quantization to networks for resource-impoverished edge devices. The classification accuracy of the method on the ESC-50 dataset is 87.1%, and the classification accuracy on the UrbanSound8K dataset is 84.45%.

However, due to cost and resource constraints, data imbalance, and data annotation problems, the training dataset may not be sufficient, resulting in a poor classification performance of deep learning models. Therefore, the use of data augmentation methods will be one of the ways to improve the classification performance. Data augmentation methods in the sound field include traditional sound signal-processing methods, such as time stretching, pitch shifting, clipping, suppression, adding noise, adding reverberation, etc. [26,27]. Data augmentation using traditional sound signal-processing methods can lead to inefficiencies. Therefore, we need simple, intuitive, and effective data augmentation methods to train acoustic models to learn to extract information from sound data. In [28], FilterAugment was proposed as a data augmentation method to regularize acoustic models in various acoustic environments. FilterAugment simulates an acoustic filter by applying different weights on frequency bands, enabling the model to extract relevant information from a wider frequency region. FilterAugment improves the performance of sound event detection models by 6.50%. In [29], SpecAugment was proposed for time warping, time masking, and frequency masking. Instead of applying data augmentation to the waveform, these processes can be directly applied to the logarithmic spectrogram. This method achieves a 6.8% word error rate (WER) in other tests without a language model and a 5.8% WER with shallow fusion with a language model. In [30], classifiers are combined that utilize standard signal augmentation (SGN), short signal augmentation (SSA), super signal augmentation (SSiA), time-scale modification (TSM), short spectrogram augmentation (SSpA), and super spectro augmentation (SuSA) data augmentation techniques to retrain five pre-trained convolutional neural networks (CNN). The experimental results show that this achieved a classification accuracy of 88.65% in the ESC-50 dataset.

## 3. Sound Classification Mechanism

In this section, a CNN-based sound classification mechanism is proposed, and a data augmentation method is used to reduce the amount of training data required for the classification model and improve the performance of the classification model. The proposed sound classification mechanism includes three parts: data preprocessing, data augmentation, and the CNN classification model.

### 3.1. Data Preprocessing

In order to enable the sound signal to become the input of the CNN classification model after being properly processed, a preprocessing procedure was performed. The preprocessing procedures include frame division, pre-emphasis, Hamming window, signal transformation, Mel filter, and discrete cosine transform (DCT), and each procedure is described as follows:

- Frame blocking: The sound signal is continuously changing. To simplify the continuously changing signal, it is assumed that the sound signal does not change in a short time scale. Therefore, the sound signal is aggregated into a unit with multiple sampling points (*N*), which is called an “audio frame”. An audio frame is 20~40 ms. If the length of the audio frame is shorter, there will not be enough sampling points in each audio frame to perform reliable spectrum calculation, but if the length is too long, the signal of each audio frame will change too much. In addition, to avoid excessive changes between two adjacent audio frames, we will allow an overlapping area between two adjacent audio frames, and this overlapping area contains about half or one-third of the sampling points (*M*) in the audio frame.
- Pre-emphasis: To highlight the high-frequency formant, the sound signal will first pass through a high-pass filter.

(1) $$S\left(n\right)=s\left(n\right)-\alpha \times s\left(n-1\right),\;\forall n\in N$$

where *s(n)* is the original sound signal, *s(n* − 1*)* is the signal after a high-pass filter, and *α* is the pre-emphasis coefficient, usually between 0.9 and 1.

- Hamming window: Suppose that the audio-framed signal is *S(n)*. To increase the continuity between the sound frames, the divided audio frames are multiplied by a Hamming window to avoid signal discontinuity in the subsequent Fourier transform. The Hamming window is shown in Formula (2), and the result of multiplying the sound frame by the Hamming window is shown in Formula (3):

(2) $$W\left(n,\;\alpha \right)=\left\{\begin{array}{cc} \left(1-\alpha \right)–\alpha \times cos\left(\frac{2\pi n}{N-1}\right), & 0\leq n\leq N-1\\ 0, & Otherwise \end{array}\right.$$

(3) $$S^{’}\left(n\right)=S(n)\times W(n,\alpha )$$

where *W(n,α*) is the Hamming window, and *n* is the *n*th sampling point. The Hamming window size produced by different α values is different. *N* is the number of sampling points, and α is generally set to 0.46.

- Signal transformation: The change in the sound signal in the time domain will continue to change over time, so the sound signal cannot be effectively discussed in the time domain. In the frequency domain, short-term speech signals appear periodic. Generally, the speech signal is converted from the time domain to the frequency domain by a discrete Fourier transform (DFT), and the characteristics of the sound signal are observed in the frequency domain, or the characteristic parameters in the frequency domain are extracted. The formula for the discrete Fourier transform is as follows:

(4) $$F\left(k\right)=\sum_{n=0}^{N-1}S^{’}\left(n\right)\times e^{-j\times \frac{2\pi nk}{N}},0\leq k<N$$

In order to reduce the number of calculations and speed up the calculation, the fast Fourier transform (FFT) will be used instead of the DFT. The formula for the discrete Fourier transform is as follows:

(5) $$F\left(k\right)=F_{even}\left(k\right)+F_{odd}\left(k\right)\cdot W_{2M}^{k},\;k=1,2,\ldots ,\;M-1$$

(6) $$\mathrm{Where}\;\left\{\begin{array}{c} \begin{array}{c} F_{even}\left(k\right)=\sum_{n=0}^{M-1}S^{’}\left(2n\right)\cdot W_{M}^{nk}\\ F_{odd}(k)=\sum_{n=0}^{M-1}S^{’}(2n+1)\cdot W_{M}^{nk}\\ W_{N}=e^{-j\frac{2\pi }{N}} \end{array} \end{array}\right.$$

- Mel filter: A set of triangular bandpass filters is selected, usually including *P* triangular bandpass filters. *P* is usually set at a value between 20 and 40. The center frequency and bandwidth of each triangular bandpass filter are determined according to the Mel scale, which is a non-linear frequency scale based on the pitch perceived by the human ear. The frequency domain response of a triangular bandpass filter is computed. The response function of each filter is a triangle, which takes the maximum value at the center frequency and then gradually decreases to the left and right sides until the frequency is 0. The response function of each filter is convolved with the spectrogram to obtain the output of each filter in the frequency domain. This output represents the logarithmic energy (*E_k_*) of the audio in this frequency band, which is equivalent to dividing the original signal into several bandpass signals of different frequencies. The Mel frequency represents the general human ear’s sensitivity to frequency, and it can also be seen that the human ear’s perception of frequency, *f,* logarithmically changes. The conversion relationship between the Mel frequency and the general frequency, *f,* is as in Formula (7):

(7) $$Mel\left(f\right)=2595\times \log\left(1+\frac{f}{700}\right)$$

- Discrete cosine transform (DCT): The above logarithmic energies, *E_k_*, are brought into the discrete cosine transform to find the *L*-order Mel-scale Cepstrum parameter, where *L* is usually 12.

(8) $$C_{m}=\sum_{k=1}^{N}E_{k}\times \cos\left(\frac{m\times \left(k-0.5\right)\times \pi }{N}\right),\;m=1,\;2,\;\ldots ,\;L$$

where *E_k_* is the value of the inner product of the triangular filter and the spectral energy calculated in the previous step, and *N* is the number of triangular filters.

**Figure 1:**
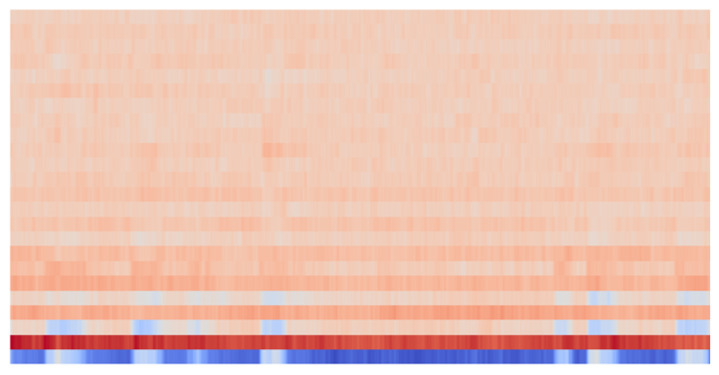
Spectrogram of a sound signal.

An example of a spectrogram that can be obtained after preprocessing the sound signal is shown in Figure 1.

### 3.2. Data Augmentation

The classification efficiency is limited by the amount of training data required for the solid classification model. Additionally, the collection and processing of training data are not very easy. Therefore, the use of data augmentation methods will help to improve the performance of sound classification. Consider the Mel filter, which is designed based on the characteristics of the frequency response of the human auditory system to sound. Since the perception of the human ear is on a logarithmic scale, the logarithmic transformation can better simulate the human ear’s perception of sound. Using a set of Mel filters, the speech signal can be divided into several different frequency bands, and the strength of each frequency band can be represented by a logarithmic value. These logarithmic values are often used as acoustic features for classification and modeling in tasks such as speech recognition. Using different numbers of triangular bandpass filters for the same sound signal will produce a similar but not identical logarithmic energy. Therefore, if *K* sets of triangular bandpass filters with different numbers are set, the same sound can produce *K* times similar sound characteristics, in order to achieve the purpose of data augmentation. In this study, the set of triangular bandpass filters and the number of triangular bandpass filters are listed in Table 1. When *K* = 1, only one set of 40 triangular bandpass filters is used to process the signal with a Mel filter. This is the original Mel filter approach, and the data are not augmented. When *K* = 2, two sets of triangular bandpass filters with numbers of 30 and 40 are used, respectively, to process the signal with the Mel filter, and the data are augmented to obtain twice the amount of signal data. Similarly, when *K* = 5, use 5 sets of triangular bandpass filters with numbers 20, 25, 30, 35, and 40, respectively, to process the signal with the Mel filter, and the data are augmented to obtain 5 times the amount of signal data.

### 3.3. Sound Classification Model

**Figure 2:**
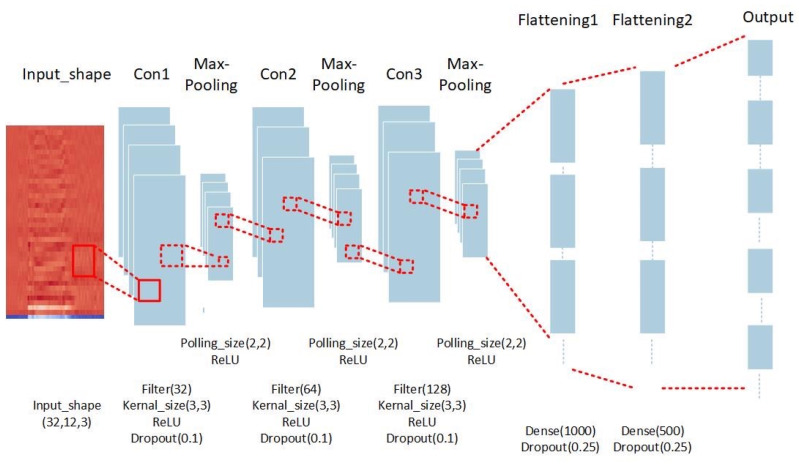
The sound classification model using CNN.

The sound classification model is a learning model using CNN, as shown in Figure 2. The input layer uses an image 40 × 173 pixels in size as the input layer, and a total of 3 hidden layers (including the convolutional layer and the pooling layer) are used, and the number of convolution kernels for each convolutional layer is 64, 128, and 256. The pooling layer uses the maximum pooling operation, and the activation function used is the Rectified Linear Unit (ReLU), in order to prevent overfitting problems during model training convergence. We added Dropout to each hidden layer to reduce overfitting, and finally classified the data through the output layer.

The operation steps of the proposed sound classification model are as follows:

Step (1): The image after the preprocessing of the data is set to a 40 × 173-pixel image as the input data of the input layer.

Step (2): This is the first convolution layer. The convolution kernel of this layer is set to 2 × 2, the feature map is set to 64, and the activation function is ReLU. The convolutional layer-processing method is similar to the image-processing method. Using the sliding-window calculation, by giving different weight combinations to the “convolution kernel”, it is possible to detect the edges and corners of the shape, and it also has the effect of removing noise and sharpening, as well as extracting these features as the basis for identification.

Step (3): This step is a pooling layer using maximum pooling. The size of the pooling layer is set to 2 × 2. The pooling layer is a method of compressing images and retaining important information. The sampling method is the same as for sliding windows, but maximum pooling is generally used. If the sliding-window size is set to 2 and the “stride” is also set to 2, the amount of data will be reduced to a quarter of the original, but because the maximum value is taken, it still retains the greatest possibility of local range comparison. That is, the pooled information is more focused on whether there are matching features in the picture, rather than “where” these features exist in the picture. Therefore, if the image is shifted, it can still be recognized.

Step (4): This is the second convolutional layer, which performs convolution operations on the data again to find various features in the image that are more detailed. The convolution kernel of this layer is set to 2 × 2, the feature map is set to 128, and the activation function is ReLU.

Step (5): This step is a pooling layer using maximum pooling. The size of the pooling layer is set to 2 × 2.

Step (6): This is the third convolutional layer, which performs convolution operations on the data again to find more detailed features in the image. The convolution kernel of this layer is set to 2 × 2, the feature map is set to 256, and the activation function is ReLU.

Step (7): This step is a pooling layer using maximum pooling. The size of the pooling layer is set to 2 × 2.

Step (8): This step consists of two flattening layers, and each node is formed into a fully connected layer to form a classifier. This means that the feature data obtained through the convolution operation will be converted into the corresponding output classification results.

Step (9): The sound classification category is output.

## 4. Experimental Results

The settings of each procedure for data preprocessing are described as follows:

- In the frame-blocking procedure: Since the sound signal is extracted at a sampling frequency of 44.1 kHz for 5 s of monophonic audio, the standard audio frame is set to 25 ms, and the overlapping area between audio frames is set to 15 ms. Therefore, there are *N* = 1130 sampling points in one audio frame, and *M* = 662 of them are the same as the adjacent audio frame.
- In the pre-emphasis procedure: According to Formula (1), the audio is enhanced.
- In the Hamming window procedure: According to Formulas (2) and (3), the 1103 sampling points in the audio frame are calculated.
- In the signal transformation procedure: The audio in the time domain is converted into the energy distribution in the frequency domain according to Formulas (5) and (6).
- In the Mel filter procedure: The energy spectrum is multiplied by a set of *K* triangular bandpass filters to obtain the logarithmic energy (*E_k_*) output by each filter, according to Formula (7).
- In the DCT procedure: The discrete cosine transform will be calculated according to Formula (8).

The hyperparameter settings of the sound classification model using CNN proposed in this paper are shown in Table 2. Each epoch represents the result of the entire model training “once”. This model was trained for 500 epochs, but during the training process, we added Early Stopping technology [31]. We first used a part of the training set as our validation set. At the end of each epoch, the accuracy of the validation set was calculated. If it is found that the performance on the verification set is getting worse and the verification performance exceeds our pre-set value (accuracy > 0.5 and epoch > 50), it may be that overfitting has occurred, and the training process will be terminated. The model uses categorical_crossentropy as the cross-validation method of the final classification result, the optimizer used is Adam, and the learning rate of the model is set to 0.001.

The confusion matrix is a method used to verify the classification effect [32], which is used to evaluate the performance of the classification model. True positive (*TP*) indicates that the result of the forecast data is the same as the actual data, and true negative (*TN*) indicates that the result of the forecast data is not the same as the actual data. False positive (*FP*) indicates that while the forecast data result is the same as the actual data, the true result is not the same as the actual data, and false negative (*FN*) means that while the result of forecast data is not the same as the actual data, the true result is the same as the actual data. Based on the results of the confusion matrix, the accuracy, precision, recall, and *F*1 score will be discussed separately to evaluate the proposed model’s classification performance.

The accuracy of sound classification is defined as shown in Equation (9), representing the ratio of correct classification cases to all classification cases in the classification model:

(9) $$Accuracy=\frac{TP+TN}{TP+TN+FP+FN}$$

The precision of the sound classification is defined as Equation (10); among all the predicted results, the proportion of correct results is predicted:

(10) $$Precision=\frac{TP}{TP+FP}$$

The recall rate for sound classification is defined as Equation (11), representing the proportion of predictions among all actual results:

(11) $$Recall=\frac{TP}{TP+FN}$$

The *F*1 score of sound classification is defined as Equation (12), where *P* stands for precision and *R* stands for recall, which is a comprehensive index of the two evaluation methods, where the value range is between 0 and 1, and the closer to 1 the value is, the better the classification result is:

(12) $$F1Score=\frac{2PR}{P+R}$$

The experimental results use different numbers of triangular bandpass filter sets for data preprocessing and data augmentation for two public datasets, ESC-50 [29] and UrbanSound8K [30], to show the excellent performance of the proposed method in sound classification.

### 4.1. Experimental Results Based on the ESC-50 Dataset

The ESC-50 [29] dataset consists of 5 s-long recordings organized into 50 semantical classes (with 40 examples per class) arranged into 5 major categories, as shown in Table 3. The dataset can be divided into three parts: the training set, the validation set, and the testing set. We assigned 81% of the data to the training set, which was utilized to train the model and adjust its parameters based on the training data. Additionally, 9% of the data were allocated to the validation set, allowing for fine-tuning of the model’s hyperparameters and evaluating its performance during training. The validation set plays a crucial role in selecting the best-performing model based on its performance on unseen data. Finally, the remaining 10% of the data were set aside for the testing set, remaining completely separate from the model development process. This subset was reserved for evaluating the final performance of the trained model and providing an unbiased evaluation of its generalization capabilities on unseen data.

**Figure 3:**
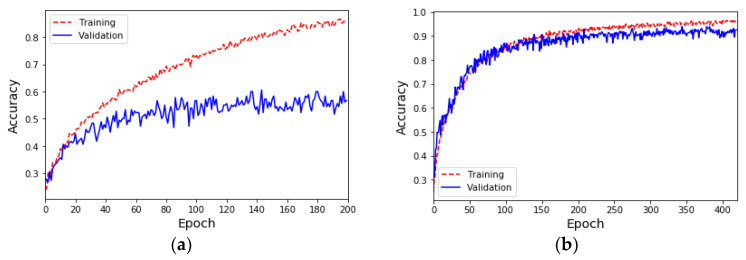
(**a**) The accuracy of the sound classification model using the ESC-50 original dataset. (**b**) The accuracy of the sound classification model using the ESC-50 dataset and amplifying the amount of data by 100% (*K* = 2).

Due to the insufficient amount of data in the ESC-50 dataset, the results of the sound classification model for the five major categories using only the original dataset (i.e., *K* = 1) are shown in Figure 3a. During the 200th training epoch, the Early Stopping mechanism was triggered to avoid overfitting problems and problems where training cannot converge. The results show that although the accuracy of the training is close to 90%, the accuracy of the validation is only about 62%. Furthermore, the results of the sound classification model for the five major categories after doubling the original data (i.e., *K* = 2) using the proposed data augmentation method are shown in Figure 3b. During the 420th training epoch, the Early Stopping mechanism was triggered to avoid overfitting problems and problems where training cannot converge. The results show that the accuracy of both the training set and the validation set is 90%. The confusion matrix and evaluation indicators for each category of sound classification using the original dataset and data augmentation are shown in Table 4 and Table 5, respectively. The results show that the data augmentation method can effectively improve the performance by 30% compared with the original dataset, meaning that the average accuracy, average precision, and average recall of classification can reach 90%, and the F1 score can reach 89%.

**Figure 4:**
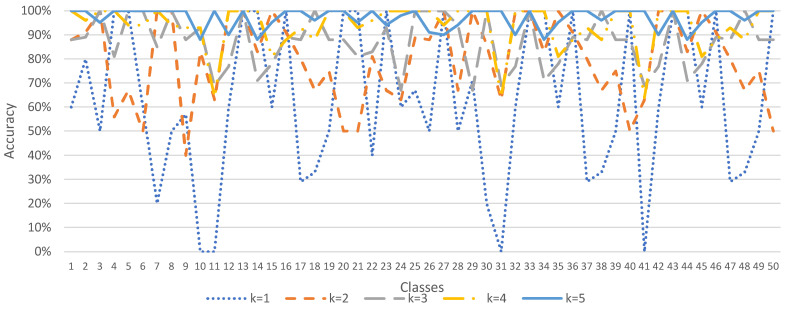
Sound classification accuracy with data augmentation in the ESC-50 dataset.

**Figure 5:**
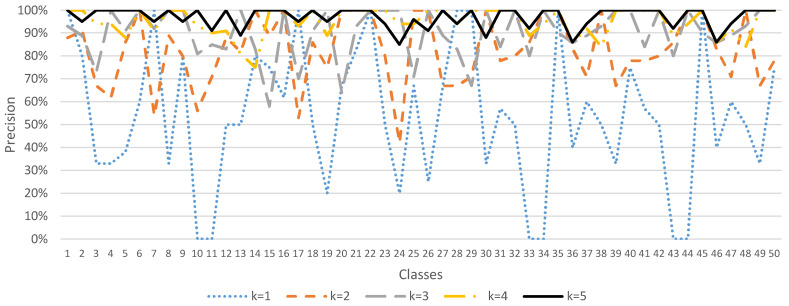
Sound classification precision with data augmentation in the ESC-50 dataset.

**Figure 6:**
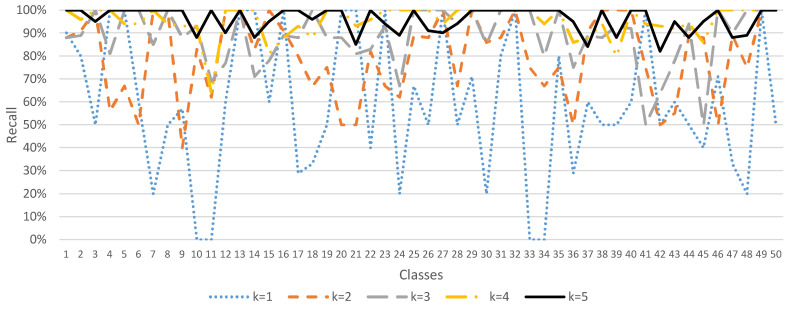
Sound classification recall with data augmentation in the ESC-50 dataset.

**Figure 7:**
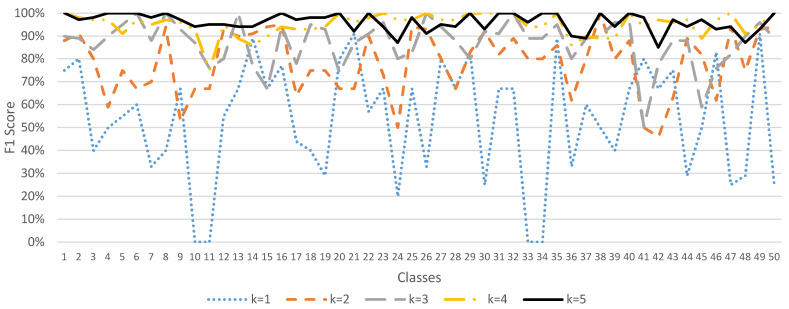
Sound classification F1 score with data augmentation in the ESC-50 dataset.

Since the ESC-50 dataset has 5 major categories, it can be subdivided into 50 semantic classes. The method of data augmentation by *K* times, where *K* = 1, 2, …, 5, was used to demonstrate the accuracy, precision, recall, and F1 score performance of the sound classification model, as shown in Figure 4, Figure 5, Figure 6 and Figure 7. In Figure 4, when *K* = 1 (data not augmented), the overall average accuracy is only 63%. The accuracy of class numbers 7, 10, 11, 17, 18, 22, 30, 31, 37, 38, 41, 47, and 48 is less than 50%. Among them, the accuracy of class numbers 10, 11, 31, and 41 is zero, showing that they cannot be classified at all. When *K* = 2, the data are doubled, and the overall average accuracy is increased to 80%. Only the accuracy of class number 9 is low, at 40%. As *K* increases to 3, 4, and 5, the overall average accuracy increases to 87%, 94%, and 97%, respectively, showing that the accuracy of each class is excellent.

In Figure 5, when *K* = 1 (data not augmented), the overall average precision is 54%. The precision of class numbers 3, 4, 5, 8, 10, 11, 19, 24, 33, 34, 36, 39, 43, 44, 46, and 49 all have less than 50%. When *K* = 2, the data are doubled, and the overall average precision increases to 83%. As *K* increases to 3, 4, and 5, the overall average precision increases to 90%, 95%, and 97%, respectively, showing that the precision of each class is excellent.

Similarly, in Figure 6, when *K* = 1 (data not augmented), the overall average recall is 59%. There are a certain number of classes (class numbers 7, 10, 11, 17, 18, 24, 30, 33, 34, 36, 47, and 48) with low recall, indicating that the classification performance is bad. However, as *K* increases to 2, 3, 4, and 5, the overall average recall increases to 80%, 88%, 95%, and 96%, respectively, showing that the recall of each class is excellent.

In Figure 7, when *K* = 1 (data not augmented), the overall mean F1 score is 53%. There are a certain number of classes (class numbers 3, 7, 8, 10, 11, 17, 18, 19, 24, 26, 30, 33, 34, 36, 39, 44, 47, and 48) with a low F1 score, indicating that the classification performance is bad. However, as *K* increases to 2, 3, 4, and 5, the overall average F1 score increases to 78%, 87%, 95%, and 96%, respectively, showing that the F1 score in each class is excellent.

### 4.2. Experimental Results Based on the UrbanSound8K Dataset

**Figure 8:**
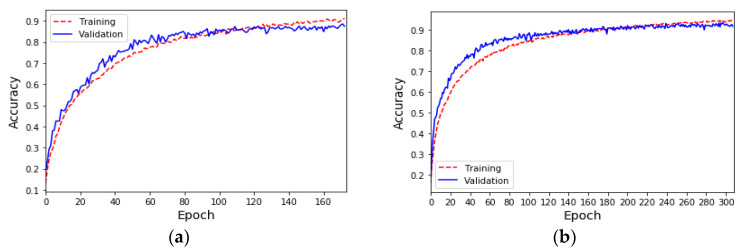
(**a**) The accuracy of the sound classification model using the UrbanSound8K original dataset. (**b**) The accuracy of the sound classification model using the UrbanSound8K dataset and amplifying the amount of data by 100% (*K* = 2).

The UrbanSound8K [30] dataset contains 8732 labeled sound excerpts of urban sounds from 10 classes: air_conditioner, car_horn, children_playing, dog_bark, drilling, enginge_idling, gun_shot, jackhammer, siren, and street_music. The length of each sound excerpt is 5 s. The amount of data in the 10 classes in the UrbanSound8K dataset is shown in Table 6. Due to the sufficient amount of data in the UrbanSound8K dataset, the results of the 10-class sound classification model using only the original dataset (*K* = 1) are shown in Figure 8a. In the 500 epochs of training, the Early Stopping mechanism was triggered to stop training at the 173rd epoch, and the accuracy of the training and the validation sets was very similar and reached 90%. In addition, the results of the sound classification model for the 10 classes after doubling the original data (*K* = 2) using the proposed data augmentation method are shown in Figure 8b. During the 308th training epoch, the Early Stopping mechanism was triggered to stop training. The results show that the data augmentation method can only slightly increase the classification accuracy by 2%, compared with the original dataset when the original dataset has sufficient data. The evaluation indicators for each class of sound classification using the original dataset and data augmentation are shown in Table 7. The average accuracy, average precision, average recall, and average F1 score of classification can reach 92%.

**Figure 9:**
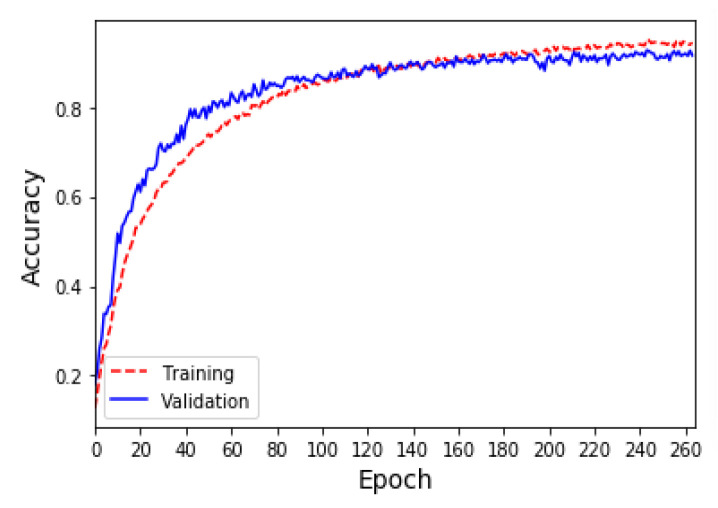
The sound classification results using this new training set.

Half of the data for each category were extracted from the UrbanSound8K dataset (that is, the number of data was 4366), and the data augmentation mechanism (*K* = 2) was used to generate a new training set with a data size of 8732. The sound classification results using this new training set are shown in Figure 9. During the 272nd training epoch, the Early Stopping mechanism was triggered to stop training. The accuracy of the training and validation sets was very similar and reached 91%. The evaluation indicators for each class of sound classification using the new dataset are shown in Table 8. The average classification accuracy and F1 score were 91%, the average precision was 92%, and the average recall was 90%. The experimental results show that, in the case of sufficient data, an excellent sound classification model can be established by using only half of the training data and through the proposed data augmentation mechanism.

A comparison of the proposed sound classification with the state-of-the-art is shown in Table 9. Based on the ESC-50 dataset, when the proposed method does not use data augmentation (*K* = 1), the classification accuracy is only 63%, which is much lower than the classification accuracy rates of the other three methods of 88.65%, 83.8%, and 87.1%. This is because the ESC-50 dataset has many classes and the amount of data for each class is insufficient, which makes the classification efficiency of the proposed method using only this dataset low. When the proposed method uses data enhancement (K = 4 or 5), the accuracy can be greatly improved and exceeds 94%. Based on the UrbanSound8K dataset, when the proposed method does not use data augmentation (*K* = 1), the classification accuracy has already reached 90%, which is better than the classification accuracy of the other two methods, which are 80.3% and 84.45%. This is because the amount of data in each class of the UrbanSound8K dataset is sufficient, making the classification performance of the proposed method excellent. If data augmentation (*K* = 2) is used, the accuracy of the proposed method can be slightly increased to 92%.

## 5. Conclusions

In this paper, we proposed a sound classification mechanism based on convolutional neural networks and used the MFCC sound feature extraction method to convert sound signals into spectrograms. These spectrograms were then used as input data for the CNN model, after learning the feature extraction of the model to distinguish the category of sounds, and the number of different triangular bandpass filters in MFCCs was used for extraction as a method of data augmentation.

In the ESC-50 dataset, there were a total of 50 semantic categories, the types were complex, and the amount of data was insufficient, resulting in a sound classification accuracy of only 63% for the main category. With the proposed data augmentation method, when *K* is 5, the accuracy was effectively increased to 97%. It can be seen that due to cost and resource constraints, it is impossible to obtain a sufficient number of datasets, and the proposed data augmentation method can be used to provide sufficient data to establish a good classification mechanism. It is worth mentioning that the proposed data augmentation method will convert the same sound data into multiple similar but different spectrograms, so that the augmented data can be directly applied to solve the challenge of data imbalance and data labeling.

In the UrbanSound8K dataset, the classification accuracy reached 90%, and it was slightly increased to 92% through data augmentation. This shows that, if the amount of data in the dataset is sufficient, the effect of data augmentation on improving the classification accuracy is limited. However, when only 50% of the dataset was used, along with data augmentation, the establishment of the training model was accelerated, and the classification accuracy reached 91%.

In our future work, the performance of the sound classification model will be affected by environmental noise, interference, sound overlap, and other related problems generated during the actual sound reception. Therefore, the proposed data augmentation method can be improved or modified in the future, so that the proposed sound classification can be implemented in practical applications.

## Figures and Tables

**Table 1:** The sets and the numbers of triangular bandpass filters.

Sets of Triangular Bandpass Filters	Number of Triangular Bandpass Filters
*K* = 1	40
*K* = 2	30, 40
*K* = 3	20, 30, 40
*K* = 4	20, 30, 35, 40
*K* = 5	20, 25, 30, 35, 40

**Table 2:** The hyperparameters of the sound classification model.

Hyperparameters	Values
Epoch	500
Batch_Size	100
Loss_Function	categorical_crossentropy
Optimizer	Adam
Learning Rate	0.001

**Table 3:** The major categories and semantical classes of the ESC-50 dataset.

No.	Major Categories				
	Animals	Natural Soundscapes and Water Sounds	Human, Non-Speech Sounds	Interior/Domestic Sounds	Exterior/Urban Noises
1	Dog	Rain	Crying baby	Door knock	Helicopter
2	Rooster	Sea waves	Sneezing	Mouse click	Chainsaw
3	Pig	Crackling fire	Clapping	Keyboard typing	Siren
4	Cow	Crickets	Breathing	Door, wood creaks	Car horn
5	Frog	Chirping birds	Coughing	Can opening	Engine
6	Cat	Water drops	Footsteps	Washing machine	Train
7	Hen	Wind	Laughing	Vacuum cleaner	Church bells
8	Insects (flying)	Pouring water	Brushing teeth	Clock alarm	Airplane
9	Sheep	Toilet flush	Snoring	Clock tick	Fireworks
10	Crow	Thunderstorm	Drinking, sipping	Glass breaking	Hand saw

**Table 4:** Confusion matrix using the ESC-50 dataset and the data augmentation mechanism.

Actual	Original Dataset (*K* = 1)					Augmentation (*K* = 2)				
Predict	1	2	3	4	5	1	2	3	4	5
1	23	5	2	3	2	75	6	3	0	5
2	7	26	3	1	5	0	65	3	2	4
3	3	5	26	3	3	3	2	81	1	1
4	2	4	8	23	6	0	2	4	69	7
5	0	6	6	2	25	0	0	0	0	67

**Table 5:** Evaluation indicators for each category of sound classification using the ESC-50 dataset and the data augmentation mechanism.

Category	Original Dataset (*K* = 1)				Augmentation (*K* = 2)			
	Accuracy	Precision	Recall	F1 Score	Accuracy	Precision	Recall	F1 Score
1	0.66	0.66	0.67	0.68	0.84	0.96	0.84	0.90
2	0.62	0.56	0.62	0.59	0.88	0.87	0.88	0.87
3	0.65	0.58	0.63	0.60	0.92	0.89	0.92	0.91
4	0.53	0.72	0.52	0.61	0.84	0.96	0.84	0.90
5	0.64	0.61	0.63	0.60	1.00	0.80	1.00	0.89
Average	0.62	0.63	0.62	0.62	0.90	0.90	0.90	0.89

**Table 6:** The amount of data in 10 classes in the UrbanSound8K dataset.

No.	Classes	Number of Data	No.	Classes	Number of Data
1	air_conditioner	1000	6	enginge_idling	1000
2	car_horn	429	7	gun_shot	374
3	children_playing	1000	8	jackhammer	1000
4	dog_bark	1000	9	siren	929
5	Drilling	1000	10	street_music	1000

**Table 7:** The evaluation indicators for each category of sound classification using the UrbanSound8K dataset and the data augmentation mechanism.

Class	Original Dataset (*K* = 1)				Augmentation (*K* = 2)			
	Accuracy	Precision	Recall	F1 Score	Accuracy	Precision	Recall	F1 Score
1	0.93	0.93	0.83	0..93	0.98	0.91	0.98	0.94
2	0.87	0.97	0.87	0.92	0.81	0.91	0.81	0.86
3	0.88	0.77	0.88	0.82	0.89	0.86	0.89	0.88
4	0.86	0.96	0.86	0.91	0.86	0.96	0.86	0.91
5	0.85	0.91	0.85	0.88	0.91	0.93	0.91	0.92
6	0.93	0.93	0.93	0.93	0.97	0.93	0.97	0.95
7	0.94	0.91	0.94	0.93	1.00	1.00	1.00	1.00
8	0.92	0.88	0.92	0.90	0.96	0.94	0.96	0.95
9	0.97	0.92	0.97	0.94	0.98	0.88	0.98	0.93
10	0.81	0.84	0.81	0.82	0.87	0.91	0.80	0.85
Average	0.90	0.92	0.90	0.89	0.92	0.92	0.92	0.92

**Table 8:** The evaluation indicators for each category of sound classification using the UrbanSound8K dataset and the data augmentation mechanism.

Category	New Dataset (Halve the Dataset and Double the Data Using Data Augmentation)			
	Accuracy	Precision	Recall	F1 Score
1	0.97	0.93	0.97	0.95
2	0.74	0.97	0.74	0.84
3	0.84	0.85	0.88	0.86
4	0.90	0.93	0.87	0.90
5	0.89	0.92	0.89	0.90
6	0.94	0.93	0.93	0.93
7	1.00	1.00	1.00	1.00
8	0.95	0.96	0.92	0.94
9	0.91	0.93	0.91	0.92
10	0.93	0.80	0.93	0.86
Average	0.91	0.92	0.90	0.91

**Table 9:** Comparison of the proposed sound classification with the state-of-the-art.

Dataset	[30]	[22]	[25]	Proposed Method				
				*K* = 1	*K* = 2	*K* = 3	*K* = 4	*K* = 5
ESC-50	88.65%	83.8%	87.1%	63%	80%	87%	94%	97%
UrbanSound8K	--	80.3%	84.45%	90%	92%	--	--	--

## Data Availability

The datasets used in the current study are available from the corresponding author upon request.
